# Invasive aspergillosis of pulmonary hydatid cyst

**DOI:** 10.4103/0256-4947.51824

**Published:** 2009

**Authors:** Buzdar M. S. Nabi, Kamran K. Chima, Nauman Tarif, Iltafat Sultan, Syed Taifur-ul-Islam Gilani

**Affiliations:** a From the Department of Pulmonary, Critical Care Medicine and Thoracic Surgery, Services Institute of Medical Sciences, Lahore, Pakistan; b From the Department of Pulmonology, Punjab Medical College, Faisalabad, Pakistan; c From the Department of Anesthesiology, Services Institute of Medical Sciences, Lahore, Pakistan

Pulmonary aspergillosis frequently complicates existing pulmonary cavity, which is commonly due to tuberculosis.^1^–^6^ Pulmonary aspergillosis has also been reported, though rarely, in pulmonary cavities as a consequence of the removal of a hydatid cyst.^7^–^9^ We report a case with active pulmonary hydatid disease that was co-infected with aspergillosis.

## CASE

**Figure 1:**
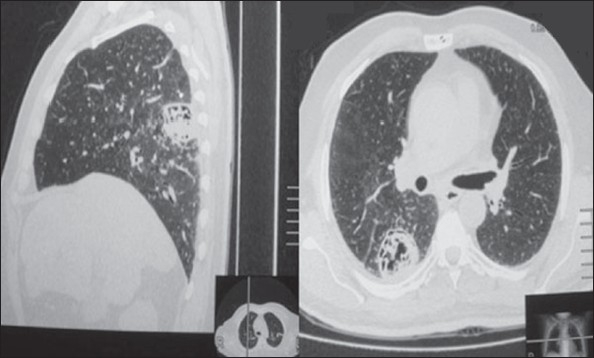
CT scan of the chest showing a cavity in the upper segment of the right lower lobe with lamellar internal echoes.

A 50-year-old male diabetic was diagnosed and treated for pulmonary tuberculosis 13 years ago. He presented with a 6-month history of low-grade fever and sweating. He also had frequent hemoptysis for the previous 3 months. He was investigated at his local hospital and referred to us for management of pulmonary aspergiloma. Examination was significant for low-grade fever and bronchial breath sounds with coarse crepitations in the right infrascapular region. A sputum smear was negative for acid-fast bacilli and grew *Aspergillus. A* plain chest x-ray showed a thick wall cavity in the upper and mid zone adjacent to the right hilum. A high-resolution CT scan revealed a large cavity in the apical segment of the right lower lobe with extensive lamellar internal echoes (Figure 1). Ultrasound of the abdomen revealed a simple hepatic cyst with no internal echoes. Bronchoscopy showed fresh blood and some necrotic material coming out of the superior segment of the right lower lobe. Culture of the material grew *Aspergillus.*

**Figure 2:**
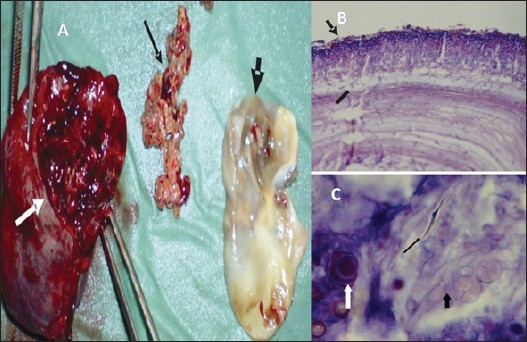
A) Gross examination of the wedge resection of upper segment of right lower lobe of the lung revealed hydatid cyst wall with necrotic material. White arrow: resected lung tissue. Thin black arrow: Fungal necrotic material. Thick black arrow: Hydatid cyst membrane. B) Thin arrow showing fungal hyphae on the outer aspect of chitinous wall of the hydatid cyst. Thick arrow showing hyphae invading the chitinous wall of the hydatid cyst. C) Echinococcus hooklets (thin black arrow) and aspergillus hyphae and spores (thick black arrow) within the cyst cavity. Calcification also seen in the cyst material (white arrow).

Wedge resection of the apical segment of the right lower lobe was performed due to persistent hemoptysis and growth of *Aspergillus* species in a possible post tuberculous cavity. Gross examination of the specimen revealed a surprisingly whitish membrane of the cyst wall with fungal necrotic material inside (Figure 2). Histopathological examination confirmed the presence of hydatid cyst along with echinoccocus hooklets with invasive *Aspergillus* within the cyst wall (Figure 2). Lung tissue around the cyst showed nonspecific chronic inflammation and an absence of fungal hyphae. The patient was treated for hydatid disease with albendazole and itraconazole for aspergillosis. The patient was free of any recurrence of either disease at the 8-month follow-up.

## DISCUSSION

Pulmonary aspergilloma frequently complicates an existing cavity that was due to tuberculosis in most cases. Nevertheless, aspergilloma can develop in any kind of pulmonary cavity, including cavities resulting from removal of a hydatid cyst.^1^–^9^ Regnard et al reported growth of aspergilloma in a post-tuberculosis cavity in 69% of a series of 89 cases with aspergilloma.^4^ Development of aspergilloma in hydatid cyst cavities is very rare. Aspergilloma in an operated hydatid cyst cavity was reported after many years in one case and after 6 months in another.^8^,^9^ Our patient is unique in that a ruptured hydatid cyst and not a post-operative hydatid cavity was secondarily infected or co-infected with aspergilloma as shown on histopathology. In fact, the aspergilloma was seen invading the wall of the ruptured hydatid cyst. We could not identify any reported case in the literature showing invasive aspergillosis in the ruptured hydatid cyst. Hemoptysis is a common presentation in ruptured pulmonary hydatid cyst and also aspergilloma, which might be the cause in our patient.^7^ We opted for wedge resection for recurrent hemoptysis since the surrounding lung tissue was healthy and because this is recommended by others.^5^

It has been reported that approximately 60% of pulmonary hydatid disease affects the right lung and 50% to 60% involves the lower lobes, which is consistent with the findings in our patient.^10^ Hydatid disease has not been reported to develop in an already existing cavity and it is unlikely that it occurred in a post-tuberculous cavity in our patient. Although it is speculative, histopathological examination did not support the presence of a cavity. Whether the hydatid cyst ruptured spontaneously or because of the invasion of *Aspergillus* is again speculative. This might be supported by the fact that *Aspergillus* species grew in the sputum prior to bronchoscopy. In our patient, a CT scan showed a lamellar pattern that might suggest hydatid cyst; however, these findings can be confused between hydatid disease and aspergilloma.^7^ Hepatic cysts also accompany 20% of the pulmonary hydatid disease cases.^10^ Hepatic cyst was also noted in our patient, but did not have the characteristic features of hydatid hepatic cyst. The presence of hydatid cyst therefore came as a surprise. Furthermore, *Aspergillus* invading the wall of an active hydatid cyst is unique in this case and has never been reported in the literature.

In conclusion, in areas where hydatid disease is still a common occurrence, pulmonary hydatid cyst should be kept in mind before labeling each cavity as post-tuberculous and aspergilloma as a secondary infection.
